# Associations of urinary enterolignans and risk of overall and cause-specific mortality with or without serum albumin adjustment: a prospective cohort study

**DOI:** 10.3389/fnut.2025.1600857

**Published:** 2025-09-25

**Authors:** Zisuo Sun, Qing Wang, Tianyi Shen, Jingming Zhu, Hongli Wang, Wanshui Yang, Zhuang Zhang, Qiang Zhou

**Affiliations:** ^1^Department of Nutrition, School of Public Health, Anhui Medical University, Hefei, Anhui, China; ^2^Key Laboratory of Population Health Across Life Cycle (Anhui Medical University), Ministry of Education of the People’s Republic of China, Hefei, Anhui, China; ^3^NHC Key Laboratory of Study on Abnormal Gametes and Reproductive Tract, Hefei, Anhui, China; ^4^Anhui Provincial Key Laboratory of Population Health and Aristogenics/Key Laboratory of Environmental Toxicology of Anhui Higher Education Institutes, Anhui Medical University, Hefei, Anhui, China; ^5^Department of Laboratory, The Second Affiliated Hospital of Anhui Medical University Economic and Technological Development Zone, Hefei, Anhui, China

**Keywords:** enterolignans, enterodiol, enterolactone, mortality risk, serum albumin

## Abstract

**Background:**

The association between enterolignans (the bioavailable metabolites of dietary lignans) including enterolactone (ENL) and enterodiol (END) and long-term risk of mortality remains limited and inconclusive. Involvement of human serum albumin (HSA) could be a possible reason behind the inconsistency. We prospectively examined the associations between urinary enterolignans and the risk of overall and cause-specific mortality among US adults and evaluated the impact of adjusting for HSA by comparing the results before and after its inclusion as a covariate.

**Methods:**

The data was obtained from the US National Health and Nutrition Examination Survey. Urinary END and ENL concentrations were measured using high-performance liquid chromatography with tandem mass spectrometric detection. Deaths from baseline until December 31, 2015 were identified through linkage to the National Death Index. Cox regression was used to estimate hazard ratios (HRs) and 95% confidence intervals (CIs) with and without HSA adjustment. Joint analysis and stratified analysis were used to evaluated the impact of HSA on the associations of enterolignans with mortality risk.

**Results:**

We documented 1,578 deaths among 10,664 participants after a median follow-up of 9.8 years. Higher concentrations of ENL were associated with lower all-cause mortality risk (comparing extreme tertiles, HR = 0.86, 95% CI: 0.74–1.00, *P*_trend_ = 0.031). However, the inverse association between urinary ENL and all-cause mortality risk became non-significant when further adjusting for HSA. Compared to individuals with low levels of both ENL and HSA, those with high levels of both ENL and HSA had the lowest mortality risk (HR = 0.71, 95% CI: 0.60–0.84). Meanwhile, urinary ENL concentrations were associated with decreased all-cause mortality risk (HR = 0.74, 95% CI: 0.59–0.93, *P*_trend_ = 0.020) only in the group with higher HSA levels.

**Conclusion:**

Adjustment of HSA attenuated the inverse association between urinary ENL and all-cause death risk to non-significance. HSA can be considered as an important covariate in the future epidemiological studies on enterolignans.

## 1 Introduction

Lignans are a large group of non-flavonoid phenolic compounds widely distributed in edible plants such as seed oils, whole-grain cereals and beans (1). Dietary lignans have recently gained considerable attention for their potential health benefits extensively studied in many *in vitro* and *in vivo* studies (2–6). There is an essential need for more epidemiological evidence to confirm the protective benefits of lignans in human populations. Most previous studies used a food frequency questionnaire (FFQ) to estimate habitual intake of plant foods, which may have been subject to measurement errors (7–9). In addition, the incomplete coverage of lignans in food composition databases may have compromised the accuracy of dietary lignan intake estimations (10). Lignans, which show individual variation in metabolism across populations, can be converted into more bioactive enterolignans including enterolactone (ENL) and enterodiol (END) by the host gut microbiota in the colon (11, 12). Therefore, some epidemiological studies used circulating enterolignans as biomarkers to reflect lignan intakes (11).

Circulating ENL and END are the most commonly used biomarkers. Dietary lignans are metabolized to ENL and END, which can passively diffuse across the enterocyte membrane (13, 14), with enhanced bioavailability and activity compared to their precursors (15). However, the association of serum or urinary ENL and END with long-term death have been less examined, with inconsistent results (16–19). Moreover, consideration of human serum albumin (HSA) could be a possible reason behind the inconsistency. Absorbed polyphenols can be stored and transported by HSA as it is the most abundant protein in plasma (20–23). The binding of lignans and other polyphenols to HSA is an important factor in determining their pharmacokinetics, pharmacodynamics and biological activities (20, 24, 25). Moreover, findings from *in vitro* studies confirm the antioxidant activity of enterolignans (15). Albumin can serve as a trap for reactive oxygen and nitrogen species due to the free thiol group of Cys34 (26). It has been reported that enterolignan binding to HSA also leads to an increase in the antioxidant activity of HSA *in vitro* (24, 27). Hence, HSA may play a role in the currently discovered health benefit of lignans. Besides, HSA levels have been reported to be correlated with circulating ENL and END (28), as well as with mortality risk (29, 30), rendering it to be a potential confounder for the relationship between circulating ENL and END with mortality. While to the best of our knowledge, few epidemiological research on lignans has considered albumin as a covariate (16). Hence, determining if HSA levels affect the association between lignans and health outcomes, thereby requiring adjustment in correlation analyses, is an important issue to address.

We hypothesized that further adjustment of HSA would influence the associations between urinary enterolignans and mortality risk. In the present study, we prospectively examined the associations between urinary enterolignans and mortality risk with or without serum albumin adjustment. Furthermore, joint analysis and stratified analysis were used to evaluated the impact of HSA on the associations of enterolignans with mortality risk.

## 2 Methods

### 2.1 Study population

Participants in our study were selected from the National Health and Nutrition Examination Survey (NHANES). NHANES consists of a series of continuous cross-sectional surveys of the civilian, noninstitutionalized US population since 1999, with a complex, stratified, multistage probability sampling design. NHANES incorporates personal interviews, physical examinations, and laboratory tests conducted by trained staff to collect nationally representative data. Further details can be found at https://www.cdc.gov/nchs/nhanes/about_nhanes.htm. For the present study, participants of the NHANES survey cycles from 1999–2000 to 2008–2010 were included. We excluded the participants who were younger than 18 years old (*n* = 26,781), had missing urinary enterolignans data (*n* = 24,641) and did not have linked mortality data (*n* = 14). Therefore, a total of 10,664 participants were included in the final analysis (Supplementary Figure 1). All participants provided the written informed consent, and the NHANES study protocol was approved by the National Center for Health Statistics (NCHS) Research Ethics Review Board.

### 2.2 Urinary enterolignans and creatinine measurement

Spot urine samples of the participants were collected at NHANES mobile examination centers (MEC) in collection cups, transferred to specimen vials and stored frozen in borosilicate glass or polypropylene vials or specimen cups. Vials were plugged by Teflon coated stoppers and sealed with an aluminum seal. Spot urine specimens were then labeled, immediately frozen to −20°C, and then shipped to the Division of Environmental Health Laboratory Sciences, National Center for Environmental Health, Centers for Disease Control and Prevention for analysis. The methods for the analysis of urine samples for END and ENL concentrations have been described in detail elsewhere.^1^ Briefly, urine samples were spiked with stable isotope-labeled internal standards to enhance method accuracy and precision. The samples were then subjected to solid-phase extraction to eliminate interferences and improve sensitivity. Finally, the samples were analyzed using negative ion mode electrospray ionization high-performance liquid chromatography–tandem mass spectrometry (HPLC-MS/MS). To adjust for the impact of glomerular filtration rate on urinary biomarker levels, urinary creatinine was determined using the Jaffe reaction on the Beckman CX3 (1999–2006) or an enzymatic (creatinase) method on the Roche ModP (2007 onwards). Creatinine-adjusted urinary concentration (μg/g creatinine) was calculated by dividing the enterolignans levels (μg/L) by the creatinine level (g/L) (11).

### 2.3 Serum albumin measurement

Serum specimens are stored under the conditions of 2–8°C, and shipped to Collaborative Laboratory Services for testing and analysis. Serum albumin concentrations were measured using the DcX800 method, a bichromatic digital endpoint method. In the reaction, serum albumin formed a complex with the Bromcresol Purple reagent. Then the change in absorbance at 600 nm was detected by the system. The content of albumin in the sample was directly proportional to the change in absorbance. More details of the serum albumin measurement process were described on the official website of NHANES (see text footnote 1).

### 2.4 Ascertainment of deaths

We ascertained mortality status via record linkage to the National Death Index (NDI) through 31 December 2015. In our analysis, cardiovascular disease (CVD) mortality was defined using the 10th revision of the International Classification of Diseases (ICD-10), including deaths from diseases of the heart (ICD-10 codes I00-I09, I11, I13, I20-I51) and cerebrovascular diseases (I60–I69). Cancer mortality was defined as code C00-C97. The NDI has been proven to be a reliable and efficient utility for ascertainment of deaths in large epidemiological studies, and over 98% of deaths can be identified using this approach (31, 32).

### 2.5 Assessment of covariates

Information on covariates was collected through questionnaires, administrated during the household interview, including demographic and lifestyle factors (i.e., age, sex, race/ethnicity, educational level, physical activity, and smoking status). Information on body weight, height, alcohol drinking status, menopausal status, use of female hormones was obtained during the MEC visit. The body mass index (BMI) was calculated as weight (kg) divided by the square of height (m^2^). Histories of diabetes and hypertension were defined according to self-reported medical diagnoses of these diseases or use of prescribed medications due to these diseases. The participants with a fasting glucose of 126 mg/dL or greater were also defined as diabetic patients. Hypertension (a systolic blood pressure *≥*140 mmHg or a diastolic blood pressure *≥*90 mmHg) was also identified through physical examination in the MEC. Abnormal liver function is defined by elevated levels of alanine aminotransferase (ALT) and aspartate aminotransferase (AST) that exceed the upper limits of the established normal range (ALT and AST: 0–40 U/L) (33). Estimate glomerular filtration rate (eGFR) was calculated according to the Chronic Kidney Disease-Epidemiology Collaboration (CKD-EPI) equation (34). Declined renal function was defined as eGFR <60 mL/min per 1.73 m^2^ (35).

### 2.6 Statistical analysis

All analyses incorporated appropriate sampling weights, stratification, and clustering of the complex sampling design to ensure nationally representative estimates. Cox regression models were used to calculate hazard ratios (HRs) and 95% confidence intervals (CIs) of death according to tertiles of enterolignan concentrations. HRs of death risk for each 1-standard deviation (SD) increase in concentrations of END and ENL were also calculated. Since dietary lignan intake and circulating enterolignan levels may differ between men and women (36, 37), urinary enterolignan concentrations were divided into sex-specific tertiles. Sex, age, race/ethnicity, education, marital status, ratio of family income to poverty, physical activity, smoking status, alcohol drinking status, body mass index, diabetes, hypertension, abnormal liver function, declined renal function, menopausal status, use of female hormones, urinary creatinine, and total energy intake were adjusted in the model 1. HSA was further adjusted in the model 2. We removed continuous covariates with missing values. For each categorical covariate in the models, we created a missing value indicator. We compared the distribution characteristics of the above-mentioned covariates between study population and overall population. Linear trend was tested by treating each exposure as a continuous variable in the models. We used restricted cubic splines to test the potential non-linear relationships between enterolignan concentrations and death risk. We also examined joint associations of enterolignan levels and HSA on mortality risk. According to the median values of enterolignan and HSA concentrations, participants were divided into four groups: participants with low enterolignans and low HSA levels, with low enterolignan and high HSA levels, with high enterolignan and low HSA levels, and with high enterolignan and high HSA levels. Using the first group as the reference group, and the Cox proportional hazards model was employed to calculate HRs for the other three groups. In stratified analysis, participants were stratified into two groups based on median HSA concentrations: participants with lower HSA levels and those with higher HSA levels. We examined the association of enterolignan levels and death risk in each subgroup. Interaction was tested using Wald test by evaluating whether the cross-product term between albumin and enterolignan levels was statistically significant. All statistical analyses were performed using R version 4.2.0 and statistical significance was defined as a two-tailed *p* value < 0.05.

## 3 Results

### 3.1 Baseline characteristics of participants

Among 10,664 participants aged 18–85 years (mean age, 46.6 years; SD, 19.6 years), we documented 1,568 deaths including 343 CVD-specific deaths and 349 cancer -specific deaths during a median follow-up of 9.8 years. Participants with higher urinary ENL levels were more likely to be married and non-Hispanic White, were older, were better educated, had a higher ratio of family income to poverty and energy intake, were more physically active, were more likely to be current drinkers, were less likely to be current smokers, had lower BMI. Similar trends were observed among participants with higher urinary END levels (Table 1). The median concentrations of ENL and END were 341.9 μg/g creatinine (interquartile range, IQR: 99.8–836.7 μg/g creatinine) and 38.5 μg/g creatinine (IQR: 14.7–91.6 μg/g creatinine), respectively. After comparing the covariate distributions of the overall population, we found no significant difference, indicating the representativeness of our sample (Supplementary Table 1).

**Table 1:** Age-adjusted characteristics of participants according to urinary enterolignans concentrations in NHANES (1999–2010)^a^_._

Characteristic	Enterolactone			*P*	Enterodiol			*P*
	Tertile 1	Tertile 2	Tertile 3		Tertile 1	Tertile 2	Tertile 3	
No. of participants	3,553	3,554	3,554		3,547	3,549	3,547	
Age, years	42.9 (18.4)	45.3 (19.6)	51.7 (19.8)	<0.001	43.7 (19.1)	46.7 (19.8)	49.4 (19.4)	<0.001
Female^‡^, %	52.3	51.4	52.3	/	51.7	51.8	52.0	/
BMI, kg/m^2^	29.5 (7.3)	28.6 (6.3)	27.2 (5.8)	<0.001	28.9(7.0)	28.7(6.6)	27.8(6.1)	<0.001
Total energy, kcal/d	2090.3 (986.8)	2087.9 (953.4)	2100.8 (899.5)	0.001	2044.3 (970.5)	2100.7 (924.3)	2131.7 (959.3)	0.202
Race/ethnicity, %				<0.001				<0.001
Mexican American	20.6	22.4	22.6		23.5	21.5	19.4	
Other Hispanic	7.2	6.3	5.9		7.9	5.7	5.8	
Non-Hispanic White	44.6	44.9	51.7		39.6	49.0	53.6	
Non-Hispanic Black	22.5	23.0	15.7		25.6	20.4	15.4	
Other race	5.2	3.4	4.1		3.4	3.4	5.8	
Marital status, %				<0.001				<0.001
Married	52.5	55.6	61.2		53.1	57.9	58.0	
Widowed/divorced/separated	22.7	21.1	17.4		22.1	19.5	19.3	
Never married	21.0	20.4	18.4		21.2	19.5	19.4	
Education, %				<0.001				<0.001
*≤*12th grade	33.5	33.6	27.0		37.3	29.8	26.5	
High school graduate/GED or equivalent	26.6	25.0	21.6		25.6	25.5	22.2	
More than high school	39.8	41.3	51.4		37.0	44.6	51.1	
Physical activity, METS-h/week				0.002				0.002
<8.3	44.4	43.8	37.1		45.8	39.8	38.9	
8.3–16.7	11.5	12.8	13.5		12.0	13.0	13.0	
>16.7	43.5	43.1	49.0		41.9	46.8	47.6	
Ratio of family income to poverty				<0.001				<0.001
<1.3	31.6	29.6	24.1		33.4	27.2	24.1	
1.3–3.5	35.1	35.5	33.5		34.6	36.1	33.5	
≥3.5	24.3	26.5	34.5		22.9	28.5	34.4	
Smoking, %				<0.001				<0.001
Never smoking	44.0	49.2	52.1		46.4	48.8	49.5	
Former smoking	22.6	22.7	24.4		21.9	22.9	24.6	
Current smoking	25.2	19.8	15.0		23.4	20.0	17.5	
Drinking, %				<0.001				<0.001
Never drinking	27.2	26.3	23.7		27.6	25.9	22.7	
Low to moderate drinking	22.1	23.7	25.7		22.8	23.5	25.4	
Heavy drinking	35.0	34.5	35.7		34.4	34.9	36.6	
Serum albumin, g/L	42.4 (3.7)	42.4 (3.8)	42.7 (3.8)	0.240	42.4 (3.7)	42.5 (3.8)	42.6 (3.9)	0.975
Diabetes, %	12.6	11.0	10.8	0.004	11.1	11.1	11.7	<0.001
Hypertension, %	37.2	33.7	30.3	<0.001	34.7	33.4	32.6	<0.001

BMI, body mass index; GED, general educational development; METS, metabolic equivalent tasks; NHANES, National Health and Nutrition Examination Survey. ^a^Variables were adjusted for age except for age and enterolignans. Continuous variables were expressed as mean (standard deviation) according to the distribution of the variables, while categorical variables are presented as percentage. *p*-values were calculated from the one-way analysis of variance or Kruskal-Wallis test for continuous variables and χ^2^-test for categorical variables. Values of polytomous variables may not sum to 100% due to missing values or rounding. ^‡^We stratified the enterolignans into tertiles according to sex-specific values.

### 3.2 Association between urinary enterolignans and mortality

As shown in Table 2, all-cause death risk of participants with the highest tertile of urinary ENL concentrations decreased by 14% (HR = 0.86, 95% CI: 0.74–1.00, *P*_trend_ = 0.031) compared with those in the first tertile. When HSA was further adjusted, the inverse association between urinary ENL and all-cause mortality became non-significant. In the restricted cubic spline analyses (Supplementary Figure 2), we found a non-linear association between ENL and all-cause mortality (*P*_nonlinearity_ = 0.543) and ENL lost its association with all-cause mortality after 4.7 μg/g creatinine. Additionally, ENL showed non-linear associations with CVD and cancer mortality (All *P*_nonlinearity_ > 0.05). Similarly, there was non-linear correlation between END and all-cause, CVD, and cancer mortality (All *P*_nonlinearity_ > 0.05). Urinary ENL levels did not present to be associated with CVD or cancer specific mortality risk. No significant association was observed between urinary END and decreasing all-cause, CVD and cancer mortality risk, regardless of whether HSA was adjusted for in the model.

**Table 2:** HRs (95% CIs) for mortality risk according to urinary enterolignans concentrations in NHANES (1999–2010).

Urinary enterolignans concentrations	HR (95% CI)				*P*_trend_^c^
(μg/g creatinine)	Tertile 1	Tertile 2	Tertile 3	Per 1-SD increase	
Enterolactone					
All-cause mortality					
No. of deaths/Participants	484/3551	559/3555	525/3555		
Model 1^a^	1 (Reference)	0.87 (0.77–0.99)	0.86 (0.74–1.00)	0.93 (0.88–0.99)	0.031
Model 2^b^	1 (Reference)	0.89 (0.77–1.03)	0.90 (0.77–1.05)	0.94 (0.88–1.01)	0.097
CVD mortality					
No. of deaths/Participants	95/3548	134/3555	114/3555		
Model 1^a^	1 (Reference)	0.90 (0.61–1.31)	0.91 (0.62–1.36)	1.00 (0.85–1.18)	0.990
Model 2^b^	1 (Reference)	0.91 (0.61–1.36)	0.93 (0.62–1.40)	1.01 (0.85–1.20)	0.932
Cancer mortality					
No. of deaths/Participants	111/3548	119/3555	119/3555		
Model 1^a^	1 (Reference)	0.99 (0.71–1.37)	0.86 (0.63–1.17)	0.92 (0.80–1.05)	0.203
Model 2^b^	1 (Reference)	0.99 (0.70–1.38)	0.88 (0.64–1.22)	0.92 (0.78–1.07)	0.261
Enterodiol					
All-cause mortality					
No. of deaths/Participants	557/3550	533/3547	471/3546		
Model 1^a^	1 (Reference)	0.96 (0.81–1.13)	1.09 (0.95–1.27)	1.05 (0.98–1.12)	0.186
Model 2^b^	1 (Reference)	0.92 (0.77–1.09)	1.08 (0.93–1.27)	1.05 (0.97–1.12)	0.235
CVD mortality					
No. of deaths/Participants	123/3549	118/3545	99/3546		
Model 1^a^	1 (Reference)	0.97 (0.71–1.35)	1.14 (0.78–1.66)	1.08 (0.91–1.28)	0.368
Model 2^b^	1 (Reference)	0.94 (0.68–1.31)	1.13 (0.76–1.67)	1.07 (0.90–1.28)	0.436
Cancer mortality					
No. of deaths/Participants	126/3549	109/3545	112/3546		
Model 1^a^	1 (Reference)	0.77 (0.54–1.12)	0.91 (0.68–1.23)	0.95 (0.81–1.11)	0.493
Model 2^b^	1 (Reference)	0.77 (0.53–1.12)	0.92 (0.67–1.25)	0.93 (0.79–1.10)	0.393

BMI, body mass index; CIs, confidence intervals; CVD, cardiovascular diseases; GED, general educational development; HRs, hazard ratios; METS, metabolic equivalent tasks. Model 1^a^ was adjusted for sex (male, female), age (years, continuous), total energy intake (kcal/day, tertiles), race/ethnicity (Mexican American, other Hispanic, non-Hispanic White, non-Hispanic Black, and other race), physical activity (<8.3, 8.3–16.7, and >16.7 METS-h/week), ratio of family income to poverty (<1.30, 1.30–3.49, and ≥3.50), marital status (married, widowed/divorced/separated, and never married), education (*≤*12th grade, high school graduate/GED or equivalent, and more than high school), smoking (never smoking, former smoking, and current smoking), alcohol drinking (never drinking, low to moderate drinking, and heavy drinking), BMI (kg/m^2^, continuous), diabetes (no, yes), hypertension (no, yes), abnormal liver function (no, yes), declined renal function (no, yes), menopausal status (no, yes), use of female hormones (no, yes). Model 2^b^ was further adjusted for serum albumin (g/L, continuous). Values were log-transformed to approximate a normal distribution of the residuals. ^c^Linear trend test was conducted by treating enterolignans as continuous variable in the model.

**Figure 1:**
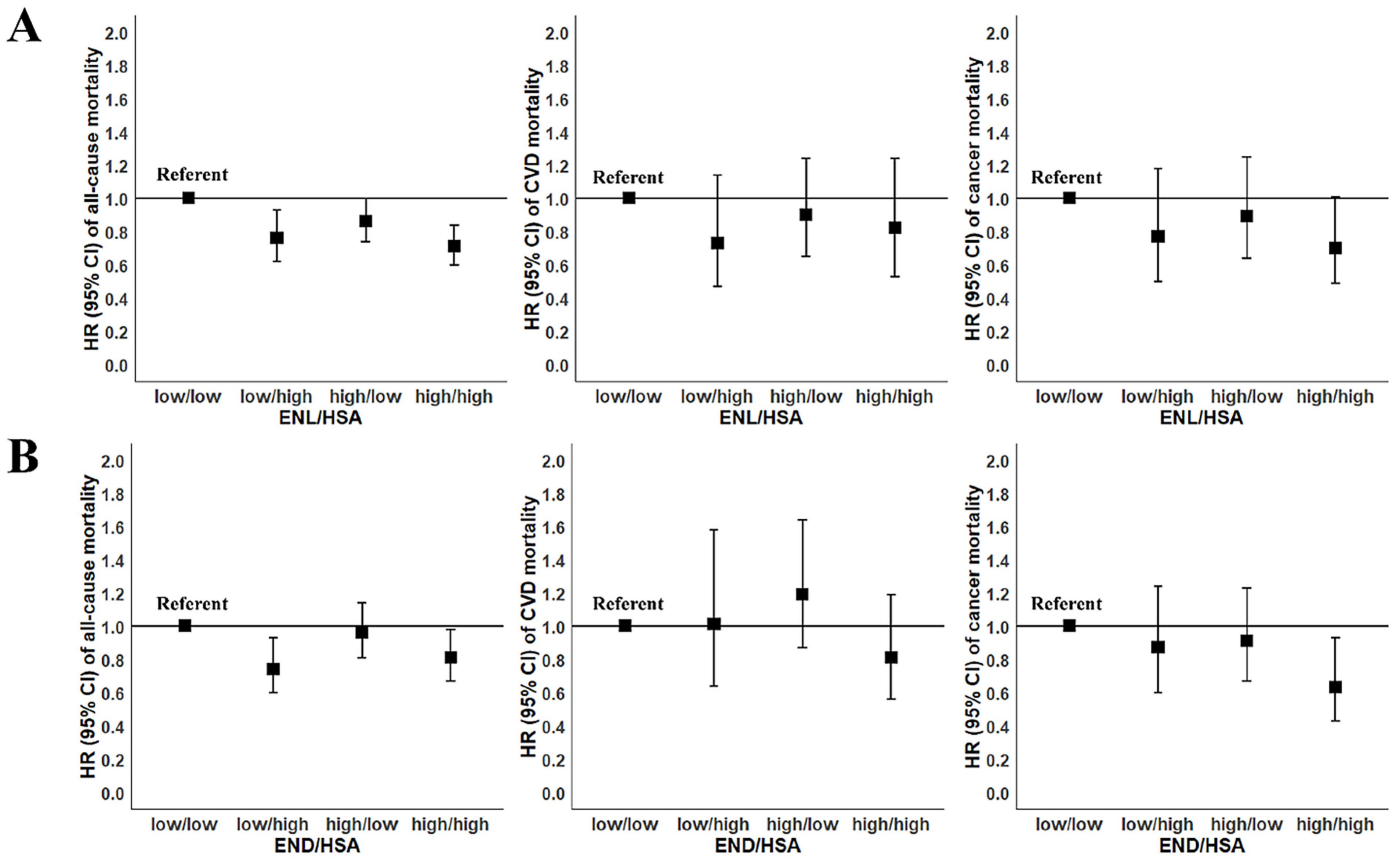
Joint association of enterolignans and human serum albumin (HSA) with risk of mortality among participants in the National Health and Nutrition Examination Survey (NHANES 1999–2010). According to the median values of enterolignans and HSA concentrations, participants were divided into four groups: participants with low enterolignans and low HSA levels (low/low), with low enterolignans and high HSA levels (low/high), with high enterolignans and low HSA levels (high/low), and with high enterolignans and high HSA levels (high/high). Using the first group as the reference group, and the Cox proportional hazards model was employed to calculate HRs for the other three groups. All models were adjusted for sex (male, female), age (years, continuous), total energy intake (kcal/day, tertiles), race/ethnicity (Mexican American, other Hispanic, non-Hispanic White, non-Hispanic Black, and other race), physical activity (<8.3, 8.3–16.7, and >16.7 METS-h/week), ratio of family income to poverty (<1.30, 1.30–3.49, and ≥3.50), marital status (married, widowed/divorced/separated, and never married), education (≤12th grade, high school graduate/GED or equivalent, and more than high school), smoking (never smoking, former smoking, and current smoking), alcohol drinking (never drinking, low to moderate drinking, and heavy drinking), BMI (kg/m^2^, continuous), diabetes (no, yes), hypertension (no, yes), abnormal liver function (no, yes), declined renal function (no, yes), menopausal status (no, yes), use of female hormones (no, yes). **(A)** The joint association of ENL and HSA with mortality risk; **(B)** the joint association of END and HSA with mortality risk. BMI, body mass index; CI, confidence interval; CVD, cardiovascular diseases; ENL, enterolactone; END, enterodiol; GED, general educational development; HR, hazard ratio; METS, metabolic equivalent tasks.

In order to further investigate the interaction of enterolignans and HSA on death risk, we performed joint analysis. The results were shown in Figure 1. Compared to individuals with low levels of both ENL and HSA (low/low group), those in all three other groups had decreased all-cause mortality risk. Participants with high levels of both ENL and HSA (high/high group) had the lowest mortality risk (HR = 0.71, 95% CI: 0.60–0.84). The all-cause mortality risk of individuals with low ENL and high HSA levels (low/high group) reduced by 24% (HR = 0.76, 95% CI: 0.62–0.93), that of participants with high ENL and low HSA levels (high/low group) reduced by 14% (HR = 0.86, 95% CI: 0.74–1.00).

**Figure 2:**
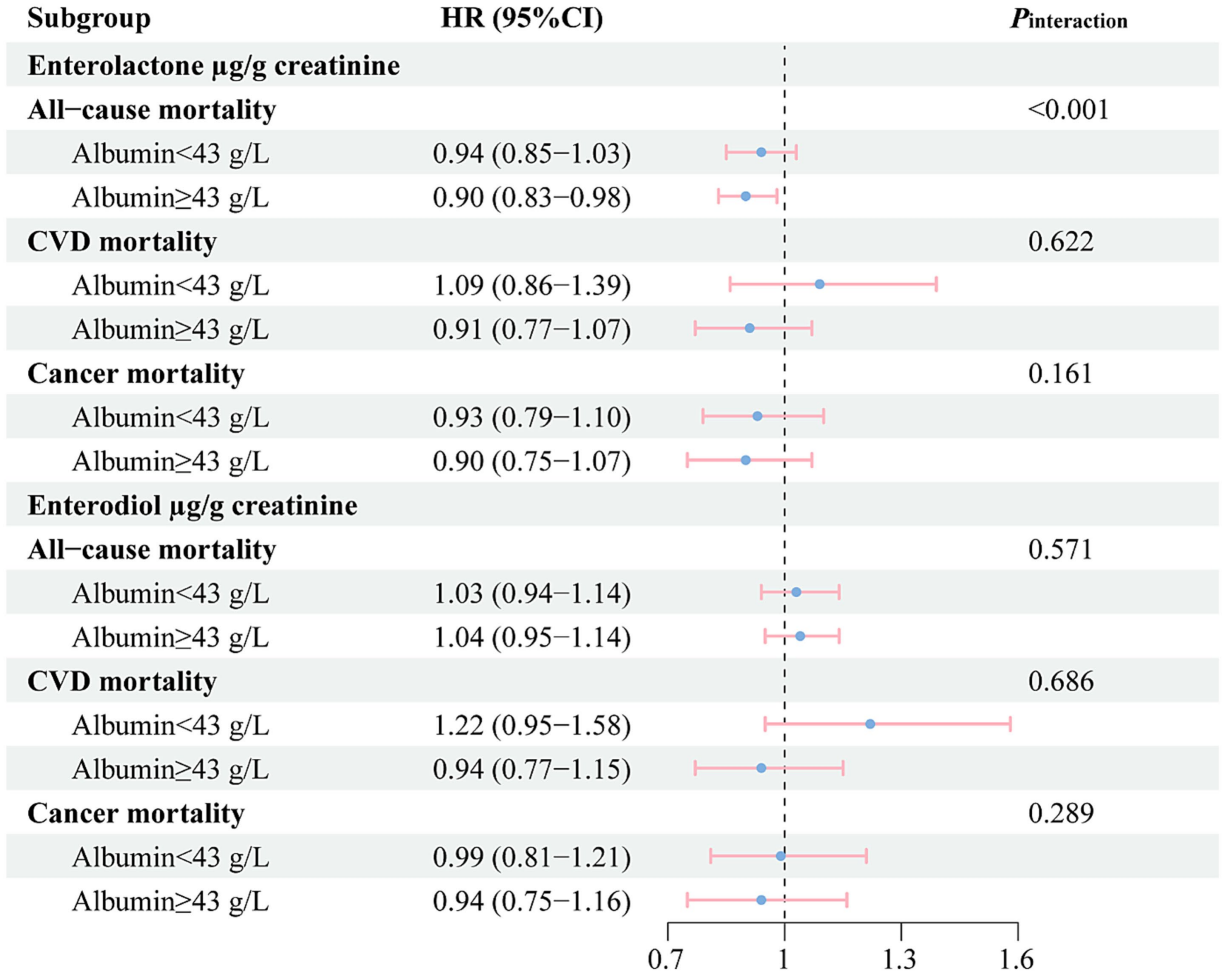
Stratified analysis for the associations between the concentrations of enterolignans (per 1-SD increase) and risk of mortality among participants in the National Health and Nutrition Examination Survey (NHANES 1999–2010). According to the median values of human serum albumin (HSA) concentrations, participants were divided into two groups: low HSA levels (Albumin <43 g/L) and high HSA levels (Albumin ≥43 g/L). All models were adjusted for sex (male, female), age (years, continuous), total energy intake (kcal/day, tertiles), race/ethnicity (Mexican American, other Hispanic, non-Hispanic White, non-Hispanic Black, and other race), physical activity (<8.3, 8.3–16.7, and >16.7 METS-h/week), ratio of family income to poverty (<1.30, 1.30–3.49, and ≥3.50), marital status (married, widowed/divorced/separated, and never married), education (≤12th grade, high school graduate/GED or equivalent, and more than high school), smoking (never smoking, former smoking, and current smoking), alcohol drinking (never drinking, low to moderate drinking, and heavy drinking), BMI (kg/m^2^, continuous), diabetes (no, yes), hypertension (no, yes), abnormal liver function (no, yes), declined renal function (no, yes), menopausal status (no, yes), use of female hormones (no, yes). BMI, body mass index; CVD, cardiovascular diseases; GED, general educational development; CI, confidence interval; HR, hazard ratio; METS, metabolic equivalent tasks. SD, Standard Deviation.

In stratified analysis (Figure 2), we found that urinary ENL levels were only correlated with a lower all-cause mortality risk among participants with higher HSA. In this subgroup, all-cause death risk of participants with the highest tertile of urinary ENL concentrations decreased by 10% (HR = 0.90, 95% CI: 0.83–0.98, *P*_interaction_ < 0.001). In the subgroup of participants with comparatively lower HSA levels, this association was not observed. Urinary ENL levels did not present to be associated with CVD or cancer specific mortality risk in both subgroups. Urinary END levels did not correlate with mortality in both subgroups.

## 4 Discussion

In this large nationally representative cohort study, our findings confirmed that further adjustment of HSA rendered the inverse association between urinary ENL and all-cause mortality marginal and non-significant. The joint effect and stratified analysis suggested that the protective role of ENL was more pronounced in individuals with higher HSA levels.

As the bioavailable metabolites of dietary lignans, the health-protective role of enterolignans have been extensively investigated by *in vitro* and *in vivo* studies (38–43). Enterolignans has been demonstrated to possess antioxidant activity in a variety of test media systems (15, 39, 40). It has been found that enterolignans could also suppress the inflammatory responses in female Alzheimer’s disease mice (41), atopic dermatitis mice (42), and an in vitro intestinal epithelium model (43). But the epidemiological studies on circulating enterolignans and mortality risk, an indicator of long-term health, are scarce with inconsistent results. A study using NHANES data (1999–2004) reported that higher urinary ENL concentrations were associated with a reduced all-cause mortality, but not with cardiovascular disease-related mortality or cancer mortality. Urinary END did not manifest any association (17). The difference in the results between ENL and END may lie in the fact that ENL is the main lignan metabolite in both urine and blood (44), as is supported by the higher urinary ENL levels than that of END in this study. Our present study expanded the sample size from 5,179 to 10,664 with consideration of more potential confounders, yielding similar results before adjustment for HSA. While another study conducted on 1,889 middle age Finnish men found an inverse but non-significant association of serum ENL with reduced all-cause mortality risk. The authors also revealed that cardiovascular death risk decreased with elevated serum ENL levels (18). Such associations were not observed in our present study and another case-cohort study of 6,065 Finnish male smokers (19). Both of these two studies were conducted in Finnish men. Differences in race, gender, dietary habits and lifestyle factors may partly explain such inconsistency (45). Additionally, results of the present study indicated that HSA levels should also be taken into consideration when assessing the associations between enterolignans and health outcomes.

In this study, we found that urinary ENL was inversely associated with all-cause mortality, while the inverse association became non-significant when HSA was further adjusted. One possible reason is that the binding of ENL to HSA may affect their health benefits. Enterolignans and other metabolites of polyphenols are known to non-covalently interact with HSA in blood through hydrophobic or hydrophilic interactions (25). Biological properties of polyphenols depend on their bioavailability. The interaction between HSA and polyphenols may influence the bioavailability of polyphenols by modulating their transport, biological activity, delivery to tissues and organs, and ultimate clearance (25, 46). This may potentially account for the potential influence of HSA on the health-beneficial properties of ENL observed in the present study. Another possibility is that, due to residual confounding, HSA may simply act as a confounder in the statistical model. HSA levels have also been proven to be negatively related with death risk among healthy participants (29, 30) and patients (47, 48). Although one cross-sectional study has reported a negative correlation between enterolignans and HSA levels among Chinese pregnant women (28), ENL concentrations were not associated with HSA levels in our study (data not shown). Hence, it is possible that the alteration in the relationship between ENL and death before and after HSA adjustment may be a consequence of the interaction of HSA with the biological activities of ENL, rather than HSA being a confounder in the model. Additionally, we cannot rule out that this attenuation reflects confounding or a statistical artifact. Further studies are needed to confirm our conclusions and above hypotheses. Moreover, RCS analysis suggested a linear dose–response relationship between ENL and all-cause mortality. The protective association for ENL became no significant at approximately 4.7 μg/g creatinine, which may suggest that ENL levels require more attention.

Our findings suggested that the association between ENL and mortality was more pronounced in participants with higher HSA levels. A potential explanation for the result could be improved antioxidant activity. Enterolactone and albumin have both been found to exhibit antioxidant properties (15, 26). Some *in vitro* studies suggested that polyphenol-albumin interaction could enhance the antioxidant activity of the complex. Binding of ENL and END to HSA could increase the reactivity of the HSA Cys34 thiol group, which plays the most important role for the antioxidant activity of HSA (24). HSA-bound quercetin was found to repair the phenoxy radical of LDL-bound tocopherol as well as the tryptophan radical of HSA (46). Almajano et al. found albumin caused a synergistic increasing antioxidant activity of green tea catechins in oil-in-water emulsions. The probable mechanism is that albumin binds with catechins and transports it to the oil–water interface, where it is highly effective at weakening the oxidation (49). This potential synergistic interaction between albumin and ENL may partially account for our observations. Albumin is an indicator of nutritional status and can be associated with function and health status (47), thus individuals with lower HSA levels might have other underlying factors affecting health, which could mask the potential inverse relationship between ENL and death. In the present study, we also observed the lowest mortality risk among participants with high levels of both ENL and HSA, suggesting that these findings are unlikely to be due to chance. However, given some potential factors, more research is necessary.

The current study has several strengths, including the use of a nationally representative sample of US adults, large sample size and reliable urinary enterolignan measurements for lignan exposure assessment. However, several limitations should be noted. Firstly, END and ENL concentrations were determined in spot urine rather than 24-h urine samples, which might introduce additional random and systematic errors because of potential circadian rhythm. However, enterolignan levels in spot urine were proven to be in good accordance with serum concentrations (50–52). Additionally, most studies utilize single-void urine enterolignans, as a biomarker of exposure. However, urinary enterolignans, measured only once, might not accurately represent habitual dietary intake, thus necessitating repeated measurements. Yet, such repeats are unavailable in NHANES due to feasibility constraints. Secondly, although we have adjusted for a wide range of potential confounders, some residual confounders due to unmeasured or inaccurately measured covariates, such as genetic susceptibility, gut microbiota, or dietary structures, cannot be entirely ruled out, and these factors may influence the associations in the study. Thirdly, since this is an observational study, we cannot determine if there is a causal relationship between urinary enterolignans and mortality risk.

## 5 Conclusion

In summary, our findings revealed that adjustment of HSA could influence the association between urinary enterolignans and mortality risk. Urinary ENL concentrations were inversely correlated with all-cause mortality only among individuals with higher HSA levels. Despite the need for more research, our work offers preliminary insights that HSA might be an important covariate to be considered in future epidemiological studies on enterolignans.

## Data Availability

Publicly available datasets were analyzed in this study. This data can be found at: The datasets generated and/or analyzed for this study can be found in the NHANES repository (https://www.cdc.gov/nchs/nhanes/.irba98.htm).

## Footnotes

^1^ https://www.cdc.gov/nchs/nhanes/about_nhanes.htm
