# Supplementary material for: Associations of urinary enterolignans and risk of overall and cause-specific mortality with or without serum albumin adjustment: a prospective cohort study
Source: Front Nutr. 2025 Sep 25;12:1600857. doi: 10.3389/fnut.2025.1600857 (PMC12509425; doi:10.3389/fnut.2025.1600857)
Supplement: Supplementary file 1 [file Table_1.DOCX]

Supplementary Material

| **Supplementary Table 1** Characteristics of the the study population and the overall population^a^ | | | | |
| --- | --- | --- | --- | --- |
|  | Study population |  | Overall population | *P* |
| No. of participants | 10664 |  | 35379 |  |
| Age, years | 46.6 (19.6) |  | 47.4 (20.1) | 0.001 |
| Female, % | 51.9 |  | 51.9 | 0.695 |
| BMI, kg/m^2^ | 28.5 (6.6) |  | 28.4 (6.6) | 0.635 |
| Total energy, kcal/d | 2095.4 (951.1) |  | 2084.6 (936.8) | 0.390 |
| Race/ethnicity, % |  |  |  | 0.556 |
| Mexican American | 21.6 |  | 21.3 |  |
| Other Hispanic | 6.4 |  | 6.5 |  |
| Non-Hispanic white | 47.4 |  | 47.5 |  |
| Non-Hispanic black | 20.5 |  | 20.5 |  |
| Other race | 4.1 |  | 4.3 |  |
| Marital status, % |  |  |  | 0.038 |
| Married | 58.3 |  | 57.0 |  |
| Widowed/divorced/separated | 20.9 |  | 21.7 |  |
| Never married | 20.7 |  | 21.3 |  |
| Education, % |  |  |  | 0.015 |
| *≤*12th grade | 31.1 |  | 32.3 |  |
| High school graduate/GED or equivalent | 24.5 |  | 24.5 |  |
| More than high school | 44.4 |  | 43.2 |  |
| Physical activity, METS-h/week |  |  |  | 0.086 |
| <8.3 | 41.6 |  | 42.7 |  |
| 8.3–16.7 | 12.7 |  | 12.4 |  |
| >16.7 | 45.7 |  | 44.9 |  |
| Ratio of family income to poverty |  |  |  | 0.228 |
| <1.3 | 30.9 |  | 31.3 |  |
| 1.3 to 3.5 | 38.0 |  | 38.4 |  |
| ≥3.5 | 31.1 |  | 30.4 |  |
| Smoking, % |  |  |  | 0.960 |
| Never smoking | 52.7 |  | 52.5 |  |
| Former smoking | 25.3 |  | 25.8 |  |
| Current smoking | 22.0 |  | 21.8 |  |
| Drinking, % |  |  |  | 0.018 |
| Never drinking | 30.0 |  | 30.7 |  |
| Low to moderate drinking | 28.4 |  | 29.5 |  |
| Heavy drinking | 41.6 |  | 39.9 |  |
| Serum albumin, g/L |  |  |  |  |
| Diabetes, % | 11.6 |  | 11.8 | 0.529 |
| Hypertension, % | 34.6 |  | 36.8 | <0.001 |

Abbreviations: BMI, body mass index; GED, general educational development; METS, metabolic equivalent tasks; NHANES, National Health and Nutrition Examination Survey.

^a^Continuous variables were expressed as mean (standard deviation) according to the distribution of the variables, while categorical variables are presented as percentage. *P* values were calculated from T test for continuous variables and χ2 test for categorical variables. Values of polytomous variables may not sum to 100% due to missing values or rounding.


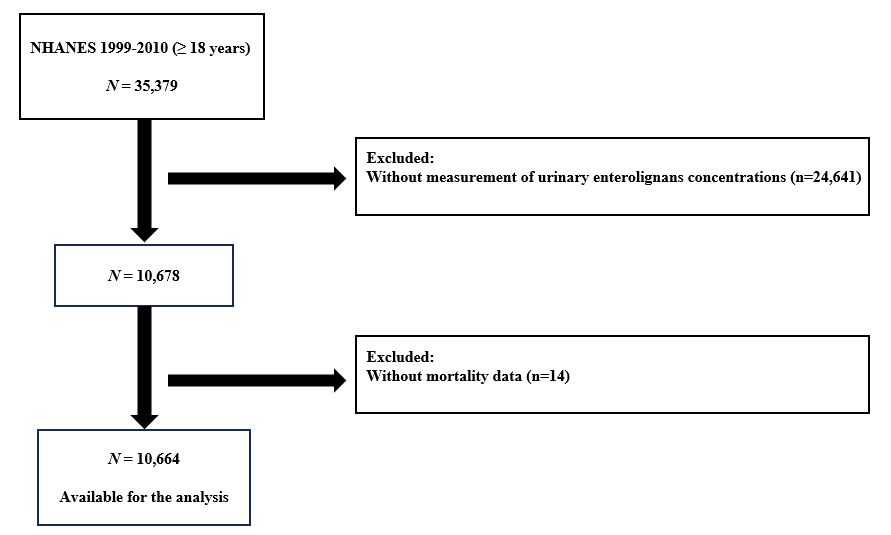


**Supplementary Figure 1** Flow chart of selection of participants in this analysis. NHANES, National Health and Nutrition Examination Survey.

**
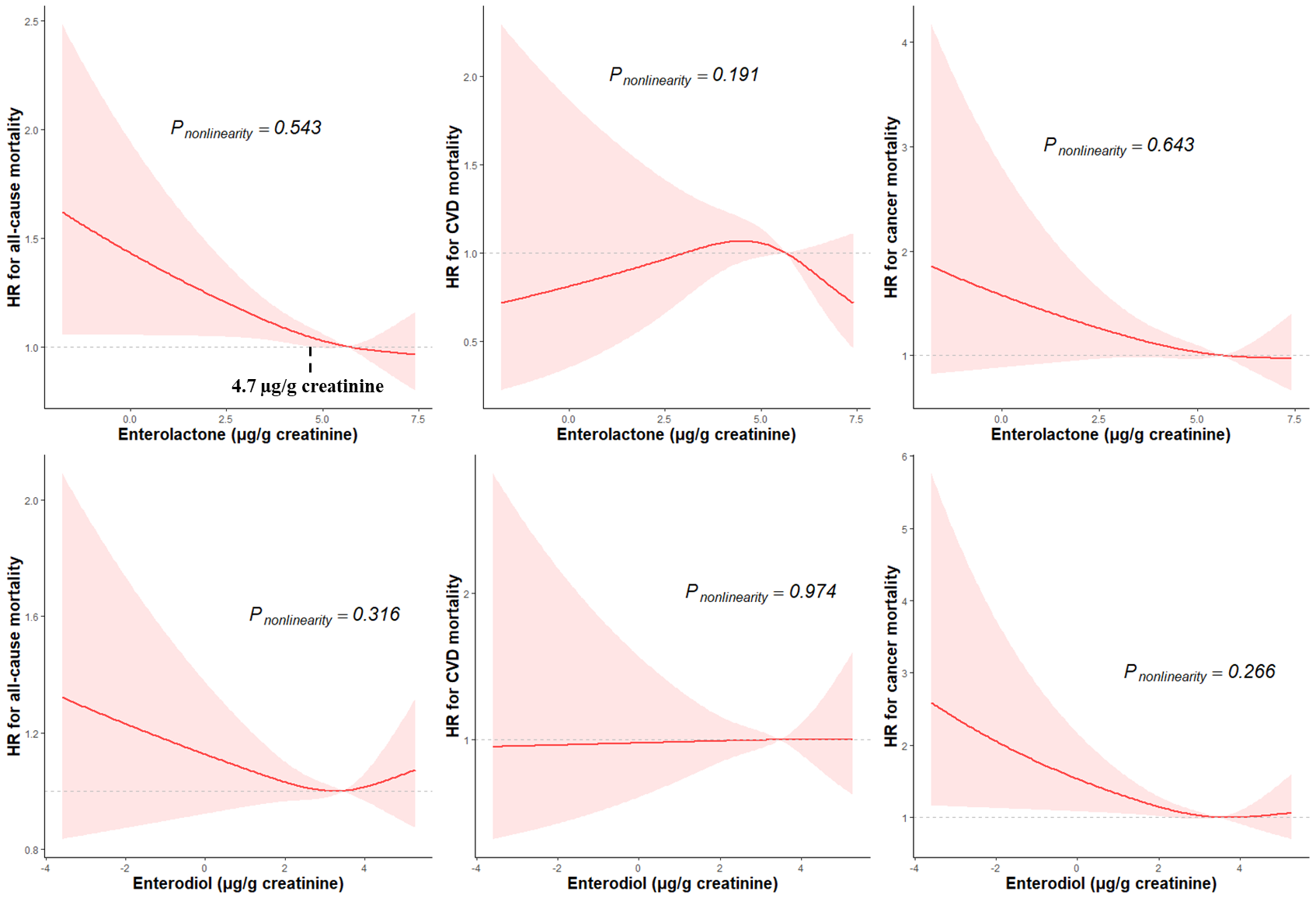
**

**Supplementary Figure 2** Associations between enterolignans and all-cause and specific cause mortality among participants in the National Health and Nutrition Examination Survey (NHANES 1999–2010) without adjustment for HSA. CVD, cardiovascular disease; HRs, hazard ratios; Covariates adjusted in the models were the same as those in Model 1 in Table 2 (see Table 2 footnote).

**
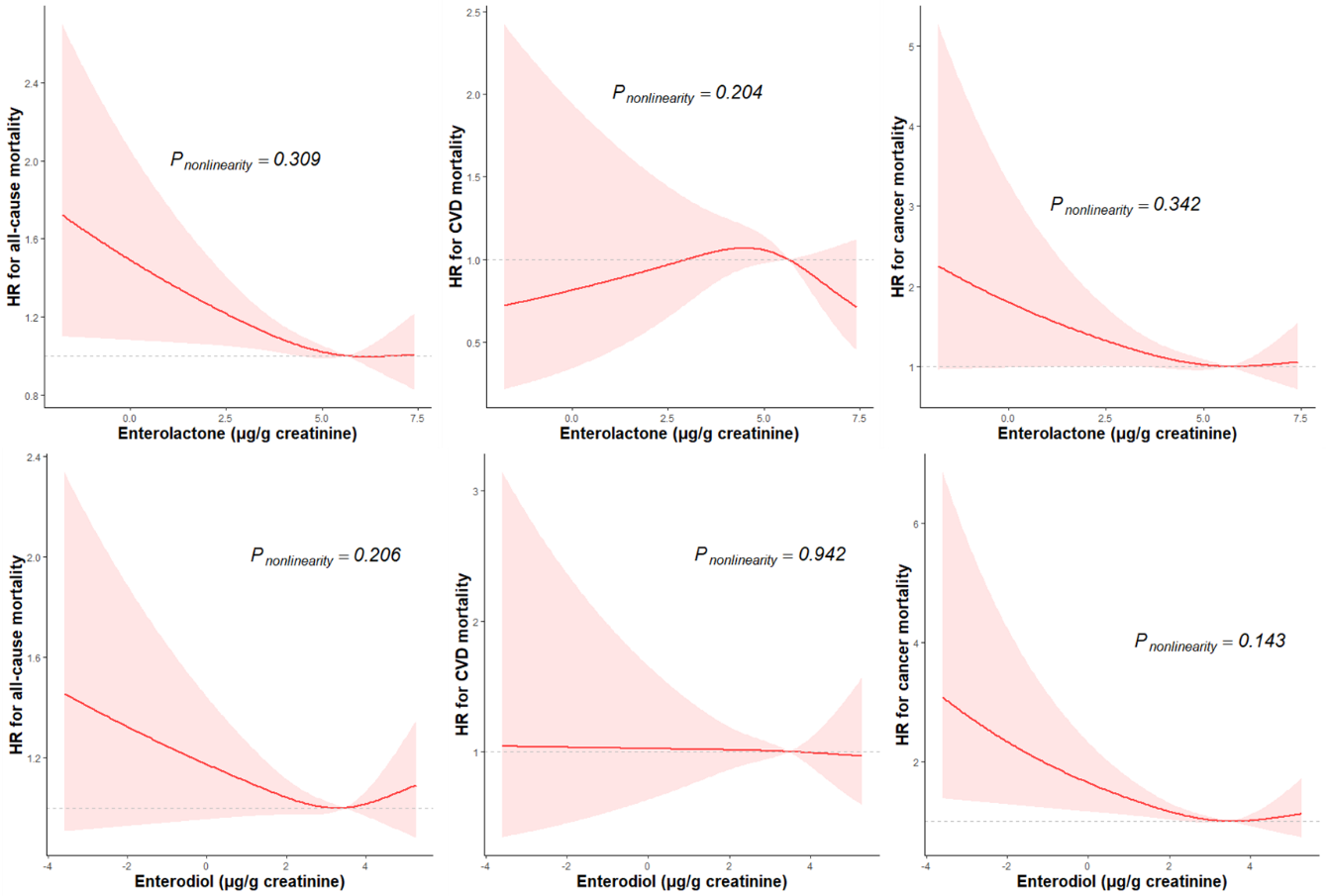
Supplementary Figure 3** Associations between enterolignans and all-cause and specific cause mortality among participants in the National Health and Nutrition Examination Survey (NHANES 1999–2010) with further adjustment of HSA. CVD, cardiovascular disease; HRs, hazard ratios; Covariates adjusted in the models were the same as those in Model 2 in Table 2 (see Table 2 footnote).
